# Fulminant lymphocytic myocarditis with unusual giant cell detection: a case report

**DOI:** 10.1093/ehjcr/ytaf248

**Published:** 2025-05-20

**Authors:** Takuma Sato, Yoshihiko Ikeda, Osamu Seguchi, Satsuki Fukushima, Yasumasa Tsukamoto

**Affiliations:** Department of Transplant Medicine, National Cerebral and Cardiovascular Center, 6-1 Kishibe-Shimmachi, Suita, Osaka 564-8565, Japan; Department of Pathology, National Cerebral and Cardiovascular Center, 6-1 Kishibe-Shimmachi, Suita, Osaka 564-8565, Japan; Department of Transplant Medicine, National Cerebral and Cardiovascular Center, 6-1 Kishibe-Shimmachi, Suita, Osaka 564-8565, Japan; Department of Cardiovascular Surgery, National Cerebral and Cardiovascular Center, 6-1 Kishibe-Shimmachi, Suita, Osaka 564-8565, Japan; Department of Transplant Medicine, National Cerebral and Cardiovascular Center, 6-1 Kishibe-Shimmachi, Suita, Osaka 564-8565, Japan

**Keywords:** myocarditis, Giant cell, Myocardial calcification, Pathological analysis, Case report

## Abstract

**Background:**

An accurate and rapid pathologic diagnostic in fulminant myocarditis is crucial for appropriate therapeutic decision-making. Since giant cells can appear in various conditions, careful judgment based on repeated imaging, pathological analysis, and close clinical follow-up is essential to make precise decisions.

**Case summary:**

A previously healthy 36-year-old woman was admitted to the hospital with haemodynamic compromise requiring mechanical circulatory support. Pathologic findings of left ventricular (LV) at the time of left ventricular assist device (LVAD) implantation confirmed the diagnosis of lymphocytic myocarditis. Cardiac function had gradually recovered, and LVAD was successfully weaned on post-operative day (POD) 10. However, surgical biopsy of LV at the time of LVAD removal revealed multinucleated giant cell formation, resulting in administered prednisolone for 2 weeks. After the withdrawal of prednisolone, pathologic findings of the right ventricle (RV) endomyocardial biopsy (EMB) specimens obtained on POD 21 showed continuous resolving myocarditis without any giant cell formation. Several additional examinations showed no evidence of an autoimmune-related disease background. The cardiac catheterisation performed on POD 85 showed normal hemodynamics with preserved LV function and EMB of the RV showed resolving myocarditis without giant cells. One year later, the patient was well without immunosuppressive therapy.

**Discussion:**

The multinucleated giant cell formation might be the accidental result of the fusion of macrophages that had ingested myocardial calcifications or degenerative macromolecules formed by the severe inflammatory process of lymphocytic myocarditis. The massive calcium deposition in both ventricles on CT and the positivity of monocytic markers in giant cells support this hypothesis.

**Learning points**

- The distinction of giant cell myocarditis (GCM) from other myocarditis is especially important.
- However, the giant cell itself is not specific for GCM and may appear under various aetiologies.
- Careful decision and repeated pathological analysis are essential when a giant cell is detected in myocarditis.

## Introduction

Fulminant myocarditis is a critical inflammatory heart disease with a distinct clinical entity. An accurate and rapid diagnostic approach is crucial for optimal management, and pathologic findings on myocardial specimens are known to be the most definitive guide for appropriate therapeutic decision-making. In contrast to the favourable prognosis of lymphocytic myocarditis, giant cell myocarditis (GCM) is a fatal disease that often results in irreversible myocardial damage requiring long-term mechanical support and/or heart transplantation. Because aggressive and long-term immunosuppressive therapy is known to improve outcomes in GCM, it is essential to differentiate GCM from other types of myocarditis in this setting.^1,2^ However, since multinucleated giant cells themselves could appear under various aetiologies,^3^ careful judgment is always required to correctly differentiate severe giant cell-related pathological disease from other types of myocarditis.

Here, we report an unusual case of acute lymphocytic myocarditis with a transient appearance of giant cells in the acute phase, which resolved spontaneously.

## Summary figure

**Figure:**
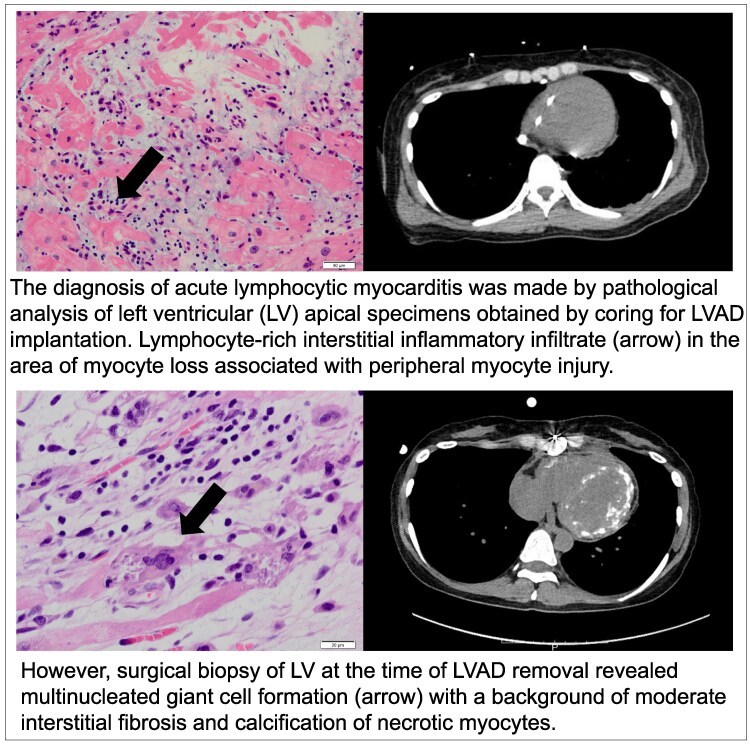


## Case summary

**Figure 1:**
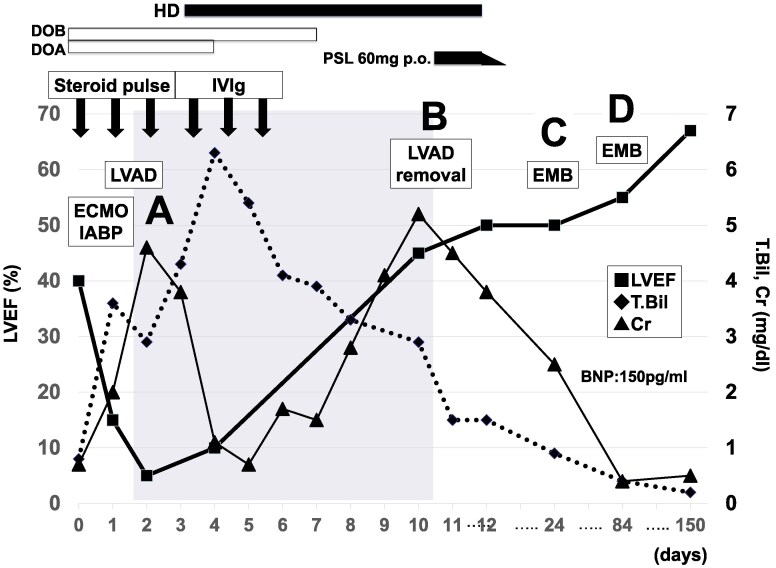
Clinical course and treatment. BNP, brain natriuretic peptide; Cr, creatinine; DOA, dopamine; DOB, dobutamine; EMB, endomyocardial biopsy; HD, haemodialysis; IABP, intra-aortic balloon pumping; IVIg, intravenous immune globulin; LVAD, left ventricular assist device; LVEF, left ventricular ejection fraction; MP, methyl prednisolone; PSL, prednisolone; T.Bil, total bilirubin.

**Figure 2:**
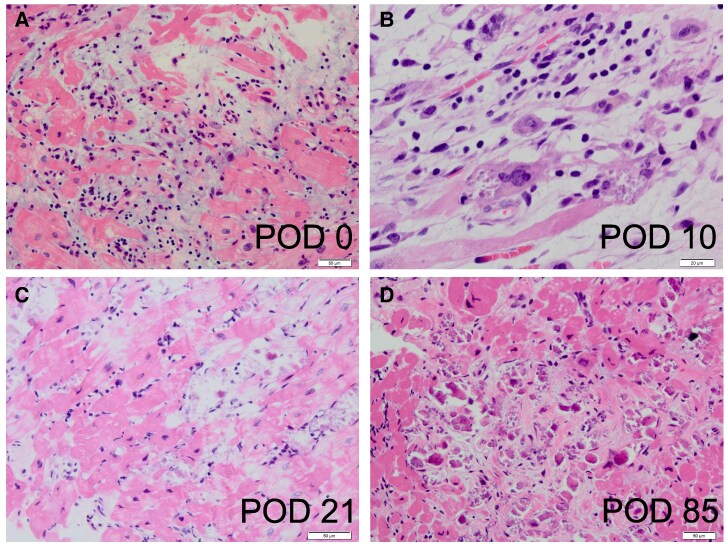
Histologic examinations. (*A*) Interstitial inflammatory infiltrate rich in lymphocytes in an area of myocyte loss associated with peripheral myocyte injury. (LV apical specimens obtained by coring for LVAD implantation). (*B*) Mixed inflammatory infiltrate with giant cells in a background of moderate interstitial fibrosis. (biopsy of LV at the time of LVAD removal/POD 10). (*C*) Calcification of necrotic myocytes. (RV EMB specimens/POD 21). (D) Resolving myocarditis with fibrosis. Inflammatory cell infiltration of interstitium without adjacent myocyte injury. (RV EMB specimens/POD 85). LV, left ventricular; LVAD, left ventricular assist device; POD, post-operative day after LVAD; RV, right ventricular.

A previously healthy 36-year-old woman was admitted to a nearby local hospital for an episode of chest discomfort with diffuse non-specific ST-T elevation on the electrocardiogram and reduced left ventricular (LV) contraction with myocardial thickening observed on echocardiography. Coronary angiography findings were normal, and she was diagnosed with myocarditis. Despite compensated hemodynamics without inotropes on the first day, the patient suddenly developed sustained refractory ventricular arrhythmias with haemodynamic compromise requiring mechanical circulatory support with an intra-aortic balloon pump (IABP) and percutaneous veno-arterial extracorporeal membrane oxygenation (VA-ECMO) the following day. The patient received intravenous methylprednisolone at a dose of 1000 mg/day for three consecutive days as steroid pulse therapy for suspected Stage D myocarditis. Despite the support of ECMO and IABP, her pulmonary congestion and organ failure worsened day by day. In addition, because of her small femoral artery diameter, she had developed severe lower extremity ischaemia due to ECMO insertion. She was transferred to our hospital for further treatment, including implantation of a left ventricular assist device (LVAD) on day 6. On admission, physical examination revealed jugular venous distension and mild peripheral oedema. In addition, the patient exhibited ischaemic discolouration of the left lower limb. Laboratory findings included elevated troponin T levels (9.860 ng/mL; normal <0.014), consistent with myocardial injury. Immediately after transfer to our hospital, the patient underwent paracorporeal pulsatile-flow LVAD implantation. Pathologic findings from the LV apex coring at the time of LVAD implantation revealed a lymphocyte-rich interstitial inflammatory infiltrate in the area of myocyte loss associated with peripheral myocyte injury, leading to the diagnosis of lymphocytic myocarditis (*Figure 2A*). Echovirus 22 antibody titres were 4 times higher in an acute phase serum than in the remission phase at 2 weeks. Viral genome detection by polymerase chain reaction was not performed on myocardial tissue. Continuous haemodialysis was introduced for 10 days for acute kidney injury. Cardiac function gradually improved under LVAD support (LV ejection fraction; LVEF 5% to 45%), and LVAD was successfully weaned on post-operative day (POD) 10 (*Figure 1*). However, surgical biopsy of LV at the time of LVAD removal revealed multinucleated giant cell formation with a background of moderate interstitial fibrosis and calcification of necrotic myocytes (*Figure 2B*). Although not all pathologic findings were typical of GCM, we could not exclude the possibility of her autoreactive involvement background leading to the development of GCM from the pathological aspect. The patient was administered prednisolone (starting at 60 mg daily) for 2 weeks. However, there were no further improvements in cardiac function.

**Figure 3:**
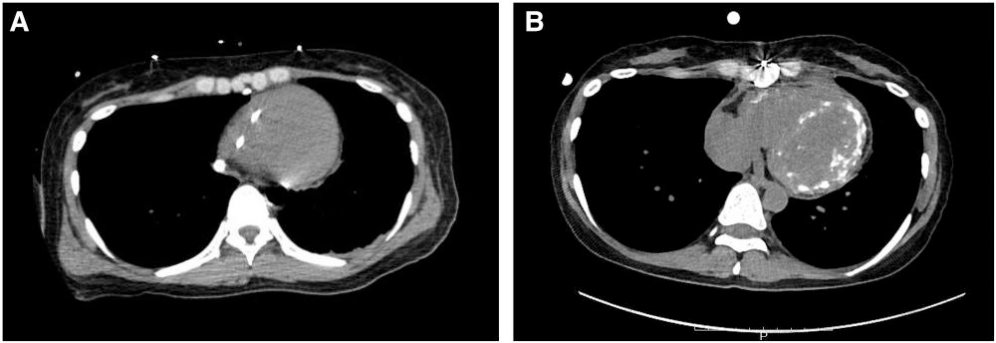
CT scan. (*A*) Before LVAD implantation. Swan-Gantz catheter in right ventricular. No apparent calcified myocardium. (*B*) Day 40 of admission. Extensive calcification in both ventricular myocardium.

After the gradual withdrawal of prednisolone, pathologic findings of the right ventricle (RV) endomyocardial biopsy (EMB) specimens obtained on POD 21 showed continuous resolving myocarditis without any giant cell formation (*Figure 2C*). Computed tomography on POD 40 showed extensive calcification in both ventricles (*Figure 3*), suggesting a severe inflammatory process associated with this condition and consistent with pathologic findings.^4–6^ Investigation of multiple viral antibody titres suggested Echovirus 22 as the cause of the virus-induced lymphocytic myocarditis. Several additional examinations showed no evidence of an autoimmune-related disease background. MRI performed on POD 53, after her renal function had improved, showed diffuse LGE in the left ventricle, which is thought to be primarily due to replacement fibrosis. Native T1, T2, ECV were elevated, consistent with the Lake Louise Criteria II (2018 revised version) for myocarditis.

The last cardiac catheterisation performed on POD 85 showed normal hemodynamics with preserved LVEF (55%), and EMB of the RV showed resolving myocarditis without giant cells (*Figure 2D*). One year later, the patient was well without immunosuppressive therapy, and she was followed up at the clinic on beta-blocker and angiotensin-converting enzyme inhibitor therapy (LVEF 68%, BNP 150 pg/mL; enalapril 2.5 mg, bisoprolol 1.25 mg). Supplementary material online, *Videos S1* demonstrates the patient's ventricular function at the time of LVAD implantation and after recovery.

## Discussion

GCM is a rare form of fulminant myocarditis associated with a poor outcome, with a reported median survival of 5.5 months from symptom onset. However, unlike lymphocytic myocarditis, survival in patients with GCM has been reported to improve with specific immunosuppressive agents, as the disease is known to be associated with autoimmune mechanisms.^7^ Even in stage D myocarditis, the immunosuppressive agent is sometimes effective in recovering native cardiac function to the point where mechanical circulatory support can be weaned off.^5^ Given this background, definitive diagnoses based on repeated myocardial biopsy are essential for the appropriate treatments of fulminant GCM. In addition, patients who survive the initial presentation of GCM may have a period of stability for about a year, followed by a second episode, and hence, it's important to have a precise diagnosis.^8^

In the current case, there are no histological findings of GCM in the initial biopsy specimens obtained by coring the LV apex for LVAD implantation. In contrast to the patchy, multifocal lymphocytic myocarditis infiltrate, GCM often diffusely infiltrates the myocardium, and the sensitivity of RV EMB for GCM is reported to be 80%.^9^ Therefore, the possibility that the large LV core specimen might have missed the giant cell was unlikely. Furthermore, the patient’s favourable clinical course without evidence of an autoimmune disease background did not suggest the possibility of GCM. However, multinucleated giant cells were unexpectedly observed in LV myocardial biopsy specimens obtained surgically at LVAD removal, making the accurate diagnosis and treatment decision difficult. While the clinical course and subsequent biopsies were not consistent with GCM, the transient presence of multinucleated giant cells raises the possibility of early or localized GCM. Because GCM was not strongly suspected clinically, only a single dose of steroids was given temporarily for 2 weeks as a diagnostic treatment, with no additional improvement in the patient's cardiac function. Further RV biopsies were performed on POD 21 and POD 85, and no giant cells were detected in either specimen. It has been reported that multinucleated giant cell itself is not specific for GCM and may appear under various aetiologies such as infection, atherosclerosis, or reaction to foreign materials (e.g. silicone, mechanical heart valves, LVAD).^10–12^ Physicians have been cautioned to pay special attention not only to giant cell formation but also to the background pathologic findings and clinical course of each patient to make a definitive diagnosis.

In this case, we did not perform genetic testing, and the exact underlying aetiology and pathogenesis of giant cell formation have remained unclear. However, from the retrospective view, the multinucleated giant cell formation in this patient might be the accidental result of the fusion of macrophages that had ingested myocardial calcifications or degenerative macromolecules formed by the severe inflammatory process of lymphocytic myocarditis. Massive calcium deposition in both ventricles on CT and the positivity of monocytic markers such as CD68 and CD163 in giant cells support this hypothesis.

## Conclusion

Accurate diagnosis is fundamental to successfully treating fulminant myocarditis, especially in cases of non-lymphocytic backgrounds, such as GCM. Since giant cells can appear in various conditions, careful judgment based on repeated imaging, pathological analysis, and close follow-up of each patient's clinical course is always essential to make precise decisions. This case underscores the need for caution when interpreting transient giant cell findings, and highlights the importance of longitudinal histopathologic follow-up in atypical myocarditis.

## Supplementary Material

ytaf248_Supplementary_Data

## Data Availability

Non-identifiable data underlying this article will be made available upon reasonable request to the corresponding author.

## Lead author biography

The author started his career in the Department of Advanced Heart Failure and Transplantation at the National Cerebral and Cardiovascular Centre in 2011. He is one of the few cardiologists in Japan with expertise in the treatment of severe heart failure, LVAD, and transplantation.

## Supplementary material

**Consent:** The patient consented to the publication of this case report in compliance with the COPE guidelines.
